# Further Evidence on the Role of Thyroid Autoimmunity in Women with Recurrent Miscarriage

**DOI:** 10.1155/2012/717185

**Published:** 2012-01-26

**Authors:** Natalia Lazzarin, Costanzo Moretti, Giovanna De Felice, Elena Vaquero, Dario Manfellotto

**Affiliations:** ^1^Fatebenefratelli Association for Research (AFaR), Ospedale Fatebenefratelli Isola Tiberina, Lungotevere de' Cenci 5, 00186 Rome, Italy; ^2^Department of Endocrinology, University of Rome “Tor Vergata”, Ospedale Fatebenefratelli Isola Tiberina, 00186 Rome, Italy; ^3^Department of Obstetrics and Gynaecology, University of Rome “Tor Vergata”, Ospedale Fatebenefratelli Isola Tiberina, 00186 Rome, Italy

## Abstract

It has been twenty years since the first paper reporting the association between thyroid antibodies (TAIs) and spontaneous miscarriage was published. Following this observation, several studies have clearly demonstrated an increased prevalence of TAI in patients with recurrent miscarriage (RM). However, the exact mechanism underlying this association remains a matter of debate. The aim of the present study was to evaluate the thyroid function, throughout a specific test, in patient with RM and TAI focusing on the hypothesis that TAI should be an indirect sign of a mild thyroid dysfunction. 46 patients with RM and TAI were included in the study. All patients underwent short TRH stimulation test showing an abnormal response in the vast majority of cases (65%). Normal FT4 and FT3 mean values were found whereas TSH values were in the upper normal range (2.64 ± 1.3 mUI/L). Our data support the hypothesis that in patients with RM the presence of TAI is an indirect sign of a subtle thyroid dysfunction detectable by a specific test. This test give the possibility to identify women with RM in which specific therapeutic approaches could effectively improve the possibility for a successful pregnancy.

## 1. Introduction

During the last decade much work has been done in the area of thyroid and pregnancy, and significant advances in the understanding of thyroid function modifications have been reached. A general agreement has developed in the potential relationship between different pregnancy pathologies, such as abortion, gestational hypertension, and diabetes, and even subclinical thyroid disorders such as subclinical hypothyroidism and the presence of thyroid antibodies (TAI) [1]. In particular, a great amount of observations have clearly established that the presence of TAI is associated with a significant increased miscarriage risk [2]. Furthermore, it has been demonstrated that the prevalence of TAI in patients with recurrent miscarriage (RM) is higher with respect to that found in normal fertile control suggesting that TAI should be considered as an independent indication of the risk of pregnancy loss [3, 4]. Nevertheless, the exact mechanism underlying the association between TAI and miscarriage remains a matter of debate, and a clear explanation for this phenomenon has not been clearly established. Effectively, two possibilities for this association can be considered: immune dysfunction or mild thyroid abnormalities. Some authors suggest that the most plausible hypothesis is that women with thyroid autoantibodies have an underlying, more generalized autoimmune activity, which leads to increased fetal losses [5, 6]. If this explanation is true, then it would be reasonable to think that the best therapy would require the modulation of the immune function. Nevertheless, to date, no studies have demonstrated the efficacy of these therapies in preventing miscarriage in patients with TAI. In addition, our previous study has demonstrated that treatment with high-dose immunoglobulin, a therapy which can influence the immune system, does not significantly improve the obstetric prognosis in patients with unexplained RM and TAI [7]. In fact, in agreement with other authors, we hypothesise that in women exhibiting TAI the thyroid is less able to adapt to the increased requirements of pregnancy leading to an inadequate thyroid hormones release [8]. From this point of view, it is plausible that the increased miscarriage rate in patients with TAI could be due to a thyroid dysfunction, rather than a generalized overreaction of the immune system. The aim of the present study was to evaluate the role thyroid autoantibodies in patients with RM focusing on the study of thyroid function throughout a specific test.

## 2. Material and Methods

### 2.1. Patients

From January 2001 to January 2010, six hundred and thirty patients with a history of RM attended the outpatient Clinic of the University of Rome “Tor Vergata”. Among these patients 46 were prospectively included in the study. Clinical inclusion criteria included the presence of 2 or more first trimester consecutive abortions. Laboratory criteria required the presence of TAI (antithyroperoxidase and/or antithyroglobulin antibodies). Patients with chronic diseases, with chronic ongoing treatments, or oral contraception were excluded from the study. Thyroid function tests included FT4, FT3, and basal and TRH-stimulated serum TSH (assessed 20 min after the TRH bolus). Clinical features of patients are summarized in Table 1.

### 2.2. Methods

A standard short TRH test was performed in early follicular phase in addition to routine hormonal checks by giving 200 mcgr TRH (TRH BIOCHEM, Ferring Gmbh, Berlin, Germany) intravenously and measuring the TSH level at 0 and 20 minutes after bolus. In all studied subjects, TRH test was performed at the 7th/8th day of the menstrual cycle after an overnight fasting at 8 AM.

Serum TSH, FT3, and FT4 were measured using a supersensitive electrochemiluminescence immunoassay (Roche, Mannheim, Germany). Thyroperoxidase antibody and thyroglobulin antibodies were evaluated by means of an electrochemiluminescence immunoassay (Roche, Mannheim, Germany). Thyroperoxidase antibody and thyroglobulin antibodies were considered positive when values exceeded 65 IU/mL for TPO-Ab and 115 UI/mL for TG-Ab. According to previous studies, impaired response was defined when TSH TRH-stimulated level was higher than 15 mUI/mL 20 minutes after the bolus [9].

## 3. Results

**Figure 1:**
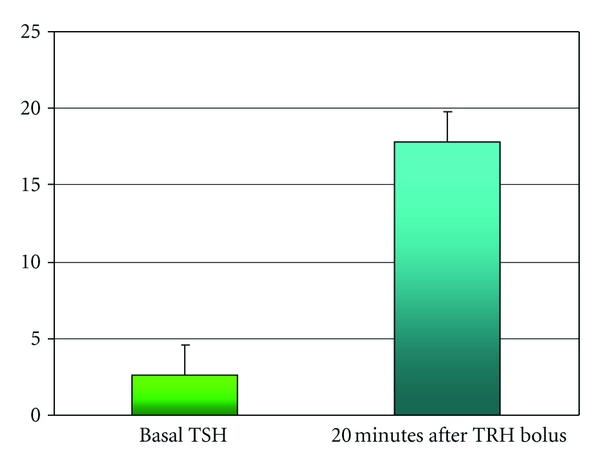
Mean basal (green) and TRH-stimulated TSH (blue) values (mUI/mL) in patients with RM and TAI.

As reported in Figure 1, the vast majority of patients showed an abnormal TRH-stimulated TSH response. In fact, 30 out of 46 patients (65%) showed a TSH TRH-stimulated level higher than 15 mUI/mL 20 minutes after the bolus with a mean TSH values of 17.8 ± 8.1 mUI/L. However, as shown in Table 1, the mean FT3 and FT4 levels were within the normal range in all cases. Although the mean TSH values resulted in the upper part of the normal range. It has note worthily been established that a TSH value below 2.5 mUI/L is advisable in women wishing to conceive [10, 11]. Finally, all patients had normal thyroglobulin serum concentrations.

## 4. Discussion

The present study supports the hypothesis that patients with RM and TAI are characterized by a subtle thyroid dysfunction that worsening during early pregnancy can lead to an inadequate thyroid hormones release. In particular, our data demonstrated that in these patients conventional laboratory tests could not reveal this abnormality. In fact, *normal *FT3, FT4, and TSH levels were found in most of the patients *with *mean levels within the normal range. Interestingly, an abnormal response to short TRH stimulation test was found in the vast majority of our patients (65%). From our point of view, this test represents an important tool for the study of thyroid function in patients with RM. In this test basal and TRH-stimulated serum TSH (assessed 20 min after the TRH bolus) are evaluated. Patients with abnormal TRH-stimulated TSH response could be considered to have an impaired thyroid function [9]. Therefore, we suppose that this exam may represent a sensible test to identify patients in which the thyroid function is less able to adapt to the increased requirement of pregnancy even in presence of normal peripheral thyroid hormones' concentrations [12]. This hypothesis is supported by a research showing that women with unsuccessful pregnancy, at the time of abortion, displayed high basal and TRH-stimulated TSH levels, with normal T4 serum concentrations, higher when compared to those observed among women with normal pregnancy [9]. Accordingly, in our previous study, we have shown that, in patients with RM and impaired response to TRH test, thyroid replacement therapy can significantly improve reproductive outcome [7]. Therefore, the high incidence of an impaired TRH test among patients with RM and TAI supports the hypothesis that these patients are characterized by a reduced functional thyroid reserve leading to an inadequate concentrations of thyroid hormones necessary for successful pregnancy. As extensively reported, thyroid hormones have a crucial role in all phases of pregnancy. In the early stage of gestation, these hormones can influence the trophoblast endocrine function through direct stimulatory effects on the production and secretion of progesterone, estradiol, hGC (*α* and *β*), and placental lactogen [13–15]. Therefore, an inadequate thyroid hormone availability at the trophoblasts level can lead to an abnormal trophoblast endocrine function [13–15]. Moreover, different studies suggest a possible role of thyroid hormones in regulating apoptosis at throphoblast level. In particular, it has been shown that thyroid hormones suppress apoptosis downregulating the expression of Fas and Fas ligand in early placental extravillous trophoblast [16]. In addition the importance of an adequate concentration of thyroid hormone for a normal placentation process is further supported by “in vitro” evidence demonstrating that thyroid can influence the invasiveness and the differentiation of cultured extravillous trophoblast cells hormones upregulating the expression of integrins and metalloproteases [17].

Later in pregnancy an impaired thyroid function has been associated with potential repercussions affecting the offspring. Recent evidence has suggested that even mild thyroid underfunction may be associated with an impaired fetal brain development. In fact, lower IQ scores have been demonstrated in children whose mothers had hypothyroidism during pregnancy in comparison with children with normal maternal thyroid function [18]. Moreover, it has been reported that children of pregnant women with normal thyroid function but increased thyroid peroxidase antibodies are at risk for impaired psychomotor development [19]. Therefore, our data supporting the hypothesis that the presence of TAI identifies women at risk to develop fetal and maternal complications suggesting that, in these patients, a therapy which can reestablish thyroid homeostasis could be essential for a successful pregnancy [7, 8]. Accordingly, in our previous study we have demonstrated that the use of thyroid replacement therapy, in patients with unexplained RM and TAI, was found to be successful in reducing miscarriages. In fact, this therapy resulted in 79% of the pregnancies ending in full-term live births, which represents a significant improvement upon their obstetric prognosis without treatment [7]. In addition, the high rate of successful pregnancy observed in the study could be due to the appropriate time of treatment initiation. In fact, the treatment was started almost one month before pregnancy in order to obtain the optimal thyroid hormone levels necessary to a normal placental development. A more recent study has confirmed the efficacy of thyroid replacement therapy in preventing miscarriage and obstetrics complications in patients with RM and TAI [8]. In this study miscarriage rate was compared between thyroid peroxidase antibody positive women who were given levothyroxine beginning in the first trimester of pregnancy to euthyroid thyroid antibody positive women who were not given levothyroxine (the control group). A statistically significant decrease in spontaneous miscarriage was seen in treated women as compared to the controls.

In conclusion, our data further support the hypothesis that in patients with RM the presence of TAI is associated with a subtle thyroid dysfunction that can be detected by specific test. In fact, the vast majority of our patients with RM and TAI showed an abnormal response to TRH stimulation tests. Therefore, the evaluation of thyroid autoimmunity and the study of thyroid functioning, through TRH stimulation tests, could be considered a useful tool for patients with a history of *RM. *Although larger studies are needed, our results suggest that these tests could identify a group of patients in which an appropriate therapy can effectively increase the possibility of a successful pregnancy.

## Figures and Tables

**Table 1:** Clinical features of patients.

Mean age (years)	35.9 ± 5
Mean abortions' number	2.5 ± 0.8
Mean abortion week	7.5 ± 1.7

Mean FT3 values (mUI/L)	3.3 ± 1.2
Mean FT4 values	4.7 ± 5.1
Mean TSH values	2.6 ± 1.8
